# Anti-*Candida* Properties of Urauchimycins from Actinobacteria Associated with *Trachymyrmex* Ants

**DOI:** 10.1155/2013/835081

**Published:** 2013-03-18

**Authors:** Thais D. Mendes, Warley S. Borges, Andre Rodrigues, Scott E. Solomon, Paulo C. Vieira, Marta C. T. Duarte, Fernando C. Pagnocca

**Affiliations:** ^1^Center for the Study of Social Insects, São Paulo State University (UNESP), 13506-900 Rio Claro, SP, Brazil; ^2^EMBRAPA Agroenergy, Parque Estação Biológica, 70770-901 Brasília, DF, Brazil; ^3^Chemistry Departament, Federal University of Espírito Santo (UFES), 29075-910 Vitória, ES, Brazil; ^4^Chemistry Department, Federal University of São Carlos (UFSCar), 18052-780 São Carlos, SP, Brazil; ^5^Department of Biochemistry and Microbiology, São Paulo State University (UNESP), 13506-900 Rio Claro, SP, Brazil; ^6^Department of Ecology and Evolutionary Biology, Rice University, Houston, TX, USA; ^7^Division of Microbiology, Center for Chemistry, Biology and Agriculture Research (CPQBA/UNICAMP), 13081-970 Paulínia, SP, Brazil

## Abstract

After decades of intensive searching for antimicrobial compounds derived from actinobacteria, the frequency of isolation of new molecules has decreased. To cope with this concern, studies have focused on the exploitation of actinobacteria from unexplored environments and actinobacteria symbionts of plants and animals. In this study, twenty-four actinobacteria strains isolated from workers of *Trachymyrmex* ants were evaluated for antifungal activity towards a variety of *Candida* species. Results revealed that seven strains inhibited the tested *Candida* species. *Streptomyces* sp. TD025 presented potent and broad spectrum of inhibition of *Candida* and was selected for the isolation of bioactive molecules. From liquid shake culture of this bacterium, we isolated the rare antimycin urauchimycins A and B. For the first time, these molecules were evaluated for antifungal activity against medically important *Candida* species. Both antimycins showed antifungal activity, especially urauchimycin B. This compound inhibited the growth of all *Candida* species tested, with minimum inhibitory concentration values equivalent to the antifungal nystatin. Our results concur with the predictions that the attine ant-microbe symbiosis may be a source of bioactive metabolites for biotechnology and medical applications.

## 1. Introduction

The increased resistance of microorganisms to antibiotics is a problem of public health [1]. The increasing number of fungal species that can infect humans, particularly immunocompromised individuals, further reinforces this concern. A limited number of antifungal agents are commercially available when compared to antibacterial drugs. This scenario motivates the search for new bioactive compounds in various biological systems using several approaches, including metagenomics and microbial genome-mining. 

Actinobacteria are widely known for their ability to produce bioactive secondary metabolites, especially compounds with antimicrobial activity. These bacteria are responsible for producing two-thirds of the commercially available antibiotics [2, 3]. Most actinobacteria species explored commercially were isolated from the soil. However, after decades of bioprospecting actinobacteria from this environment, it is becoming more difficult to obtain strains producing novel bioactive metabolites [4]. Thus, many companies have turned the search for microbial producers of novel antifungal compounds to other environments such as hydrothermal vents, marine environments, tropical rain forests, and microbial symbionts associated with plants and animals hosts [5, 6]. For example, the occurrence of actinobacteria associated with marine sponges and the fact that such strains produce compounds with antimicrobial activity confirms this potential [7–9]. In addition, endophytic actinobacteria are also explored for their capacity to produce antimicrobial compounds [10–12]. 

Several studies have focused on the association between actinobacteria and insects from an ecological perspective [13–25]. On the other hand, few studies have focused on the multitude of chemical compounds that are involved in such interactions [26]. The best studied example is the symbiosis between actinobacteria and fungus-growing ants (Hymenoptera: Formicidae: tribe Attini). In this association, the actinobacteria are found on the ants' integument and produce antimicrobial compounds that help the ants to suppress the microfungus *Escovopsis *sp. [13, 14]. This fungus is considered a specialized parasite of the ant cultivar and causes negative impacts to the ant colony [27]. 

Actinobacteria isolated from the integument of attine ants are generally classified in the genus* Pseudonocardia *and *Streptomyces. *Bioactive molecules have already been isolated and characterized from actinobacteria isolated from several attine genera [26]. *Pseudonocardia* isolated from *Acromyrmex octospinosus* and *Apterostigma dentigerum* are known to produce several compounds like (i) dentigerumycin, a complex compound active against *Candida albicans* and *Escovopsis *[28]; (ii) a nystatin-like antifungal [29]; (iii) the novel quinone pseudonocardones A–C active against the malaria causal agent *Plasmodium berghei* [30]*; *(iv) the already known antibiotics 6-deoxy-8-O-methylrabelomycin and X-14 881, both active against *Bacillus subtilis* and *P. berghei* [30]. In addition to* Pseudonocardia, *actinobacteria in the genus *Streptomyces* are also found on the integument of *Acromyrmex* workers and were shown to produce (i) candicidin, active against *Escovopsis* sp. [29, 31–34], (ii) antimycins active against *Escovopsis* sp. [32–34], and (iii) actinomycin D, actinomycin X2 and valinomycin that are active against *B. subtilis* [32]. 

Poulsen [35] suggested that the attine ant-microbe association is little explored regarding the search for new antimicrobials. The author highlights the various symbiotic associations between attine ants and microorganisms as a promising source for drug discovery, especially those with antimicrobial activity. Here, we explored the antimicrobial potential of actinobacteria isolated from the integument of* Trachymyrmex* fungus-growing ants and demonstrate the action against different medically important *Candida* species. We also report two previously described urauchimycins from a* Streptomyces* strain and emphasize the newly discovered anti-*Candida* activity of these compounds.

## 2. Material and Methods

### 2.1. Actinobacteria Isolation and Identification

Twelve *Trachymyrmex * colonies were collected in different Brazilian biomes (see Table S1 in Supplementary Material available online at http://dx.doi.org/10.1155/2013/835081). Colonies were carefully excavated in order to reach the first fungus garden chamber. Fungus garden with the tending workers and brood was sampled using an alcohol-flamed spoon and stored in sterile plastic containers. All containers were kept in a cooler during transport to the laboratory where they were maintained at 25°C.

From each colony, we randomly selected four workers for actinobacteria isolation. Then, the propleural plates were scraped with a sterile needle under a low power stereomicroscope. All ants used in the present study had a visible, whitish covering on the propleural plates. Scrapings were plated on SCN agar (in g · L^−1^: 10.0 starch, 0.3 casein, 2.0 KNO_3_, 2.0 NaCl, 2.0 K_2_HPO_4_, 0.05 MgSO_4_ · 7 H_2_O, 0.02 CaCO_3_, 0.01 FeSO_4_ · 7H_2_O and 18.0 agar supplemented with 0.05 Nystatin) [36]. After scraping, the entire body of all workers was inoculated on SCN agar. All plates were incubated at 25°C for 30 days. From each sampled *Trachymyrmex *colony, one representative strain was selected from each morphotype obtained. The strains were subcultured in YMA (in g · L^−1^: 3.0 yeast extract, 3.0 malt extract, 5.0 peptone, 10.0 glucose, 18.0 agar) and stored at −80°C in 15% glycerol.

We used a molecular approach to provide taxonomic affiliation to actinobacteria strains. Genomic DNA was extracted following the method of Sampaio et al. [37]. We carried out 16S rDNA PCR with the universal primers 27F (5′-AGAGTTTGATCATGGCTCAG-3′) and 1492R (5′-TACGGTTACCTTGTTACGACTT-3′) [38]. Reactions were conducted in a final volume of 25 *μ*L and contained 1 *μ*L of diluted DNA template (1 : 10), 2.0 *μ*L of each primer (10 mM), 2.5 *μ*L of 10X buffer, 1.0 *μ*L of MgCl_2_ (50 mM), 4.0 *μ*L of dNTPs (1.25 mM each), 0.2 *μ*L of Taq polymerase (5 U/*μ*L), and 12.3 *μ*L of ultrapure water. Amplicons were cleaned up with *GFX PCR DNA and Gel Band Purification Kit *(GE Healthcare). Forward and reverse sequences were generated using the same primers, along with an internal primer U519F (5′-CAGCMGCCGCGGTAATWC-3′). Sequences were generated using *BigDye Terminator v.3.1 Cycle Sequencing Kit* (Life Technologies) in an ABI 3130 sequencer and manually edited in BioEdit v. 7.1.3 [39]. Contigs were compared with those available in the databases NCBI-GenBank (http://blast.ncbi.nlm.nih.gov/) and Ribossomal Database Project (RDP, http://rdp.cme.msu.edu/). Sequences generated in the present study were deposited in NCBI-GenBank (accessions KC480554-KC480557).

A phylogenetic analysis was carried out in order to determine the taxonomic affiliation of strain TD025. Sequences of closest relatives were retrieved from the NCBI-GenBank and the RDP Project and aligned in ClustalW. Phylogenetic reconstruction was performed using the neighbor-joining algorithm implemented in PAUP v. 4.0 [40]. Genetic distances were calculated using the Kimura 2-parameter model of nucleotide substitution [41]. Robustness of the relationships was estimated from 1000 bootstrap pseudoreplicates. 

### 2.2. Organic Extracts and Antifungal Assays

All actinobacteria were grown in Erlenmeyer flasks (250 mL) containing 50 mL of modified MPE medium (in g · L^−1^: 5.0 soy flour, 20.0 glucose, 5.0 of NaCl, 4.0 CaCO_3_) [42]. Each flask was inoculated with five mycelium disks (1 cm in diameter) cut from a previously grown culture and then incubated at 28°C for two days on an orbital shaker at 150 rpm. From this culture, 10 mL was inoculated in two Erlenmeyer flasks (250 mL) containing 100 mL of the same medium and incubated for five days on the same conditions. After incubation, the fermented broth was separated from the mycelium by centrifugation and partitioned three times with ethyl acetate (EtOAc). The organic solvent was evaporated under vacuum, and the EtOAc extracts were diluted in RPMI-1640 culture broth containing 10% DMSO and used in the antimicrobial assays.

The antimicrobial activity of the extracts was evaluated against the yeasts *Candida albicans * CBS 562, *Candida dubliniensis * CBS 7987, *Candida glabrata * CBS 138, *Candida krusei * CBS 573, *Candida parapsilosis * CBS 604, and *Candida tropicalis * CBS 94. The minimum inhibitory concentration (MIC) was determined using the microdilution method according to the M27-A2 standard of the Clinical and Laboratory Standards Institute [43].

### 2.3. Isolation and Characterization of the Bioactive Compounds of Strain TD025

The actinobacteria strain that exhibited both a broad antifungal spectrum and lower MIC values was strain TD025. In order to identify the compounds responsible for the observed results, the strain was cultured in 5 L of modified MPE medium and the extracts were obtained as described.

The fermented broth (5 L) was separated from the cells by centrifugation and portioned three times with ethyl acetate (EtOAc). The organic solvent was evaporated under vacuum. The crude extract, a dark green oil (1.40 g), was separated by means of column chromatography using silica gel 60 eluted with n-hexane/EtOAc as the elution gradient, yielding 8 fractions. All fractions were submitted to antimicrobial assays against *C. albicans* following the procedure described above. The most active fraction (67.5 mg) was subjected to preparative TLC (thin layer chromatography) eluted with n-hexane/EtOAc (7 : 3) two times, yielding 3 subfractions. The subfractions obtained were submitted to antimicrobial assays against *C. albicans*. The most active subfraction was submitted to semipreparative HPLC separations carried out in a Shimadzu (LC-6AD apparatus, Japan) multisolvent delivery system, Shimadzu SPD-M10Avp Photodiode Array Detector, and an Intel Celeron computer for analytical system control, data collection and processing (software Class-VP). The separation was carried out using VP 250/10 NUCLEOSIL 120-5 C18 column eluted with acetonitrile/water/acetic acid (50 : 50 : 0.01) at a flow rate of 3 mL · min^−1^, yielding compounds **1** and **2**. The isolated molecules were characterized by ^1^H and ^13^C NMR spectroscopic experiments recorded on a BRUKER DRX-400 spectrometer with CDCl_3_ as solvent and TMS as internal standard.

### 2.4. Minimum Inhibitory Concentration and Minimum Fungicide Concentration of Bioactive Compounds

After isolation and determination of the structure of the targeted compounds, they were evaluated for antimicrobial activity following the method described previously. Besides the MIC determination, we also evaluated the minimum fungicidal concentration (MFC). The MFC was determined by inoculating Sabouraud dextrose medium with 10 *μ*L of the contents of each of the wells where there was growth inhibition of yeast, the MFC was defined as the lowest concentration of the substance capable of preventing the onset of colony forming units.

## 3. Results and Discussion

### 3.1. Actinobacteria Isolation and Identification

Several actinobacteria colonies were observed after incubation of isolation plates. We selected just one morphotype of each per ant colony, rendering a total of 24 strains ouf of 12 *Trachymyrmex* spp. nests (Table  S1). Four actinobacteria genera were identified and *Streptomyces *was the most abundant taxon (Table 1), corresponding to 66.67% of the strains. It is assumed that the main actinobacteria associated with the integument of attine workers is the genus *Pseudonocardia* [14–16, 20]. However, several authors have demonstrated the isolation of actinobacteria other than *Pseudonocardia* on the integument of attine ants [17–19, 21, 22, 29, 31]. The prevalence of *Streptomyces* and absence of *Pseudonocardia* among our isolates may be due to the culture medium used [44]. The SCN medium is suitable for the isolation of fast-growing actinobacteria, but according to other authors [13–16], the use of a low-nutrient medium, such as chitin agar, may provide the recovery of *Pseudonocardia* strains.

In eight out of 12 ant colonies, we obtained more than one actinobacteria morphotype (Table S1). From colony CTL080820-02, the two morphotypes isolated from two different workers were identified as the same actinobacteria species (Tables  S1 and 1). On the other hand, different actinobacteria species were isolated in the seven remaining colonies. We also observed the occurrence of different actinobacteria strains in a single worker (Tables S1 and 1). This result demonstrates the diversity of actinobacteria present on the integument of these ants (Table S1, nests SES080911-04 and SES080924-01). 

**Figure 1:**
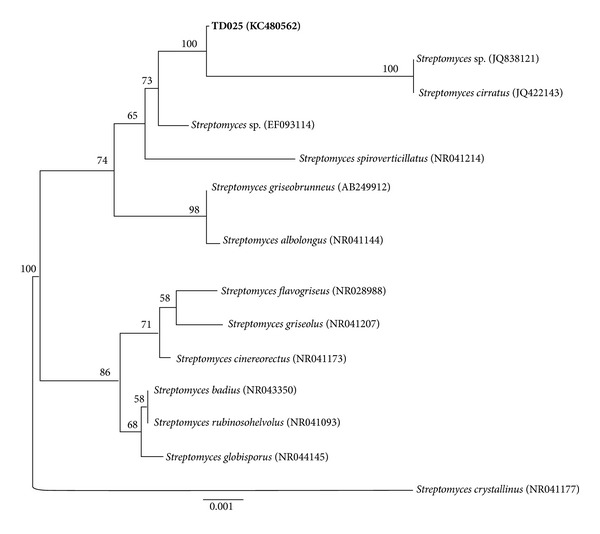
Phylogenetic relationships of strain TD025 (in bold) isolated from the integument of *Trachymyrmex* sp. The phylogeny was inferred from 16S rDNA sequences retrieved from the NCBI-GenBank using the neighbor-joining algorithm and the Kimura 2-parameter model of nucleotide substitution. Numbers in parentheses correspond to GenBank accessions. Numbers on branches indicate the bootstrap support after 1,000 pseudoreplicates. The scale bar denotes the number of substitutions per site.

The 16S rDNA sequence of strain TD025 showed 99% similarity with sequences of several species of the genus *Streptomyces* deposited in the databases. For a better characterization, we performed a phylogenetic analysis (Figure 1). The result suggests a differentiated phylogenetic position for strain TD025 when compared with the remaining sequences. This preliminary analysis allowed us to assign this strain as belonging to the genus *Streptomyces*, with *S. cirratus* as the closest relative strain (Figure 1). However, more refined phylogenetic analyses, along with morphological and physiological studies, are necessary to ensure the identification of TD025 to the species level.

### 3.2. Screening for Antifungal Activity

Our screening for antifungal activity revealed that seven out of 24 extracts (29.16%) inhibited the growth of at least one *Candida* species. *C. albicans* was the most sensitive yeast and was inhibited by seven extracts with MIC ranging between 10 and 1000 *μ*g · mL^−1^ (Table 2). The yeasts *C. glabrata* and *C. tropicalis* were the most resistant strains, being inhibited by one and two actinobacteria extracts, respectively, with MIC values of 1000 *μ*g · mL^−1^ (Table 2).

Except for strain TD034 identified as *Amycolatopsis decaplanina* (Table 1), the other actinobacteria exhibiting antimicrobial activity were identified as belonging to the genus *Streptomyces*. This genus is recognized as the largest producer of antibiotics because from approximately 3,000 known antibiotics obtained from actinobacteria, the genus *Streptomyces* contributes with 90% of this total [45].

The extracts of *Streptomyces* sp. TD025 and *Streptomyces crystallinus *TD027 showed activity against all yeast strains except for *C. glabrata*. These extracts were effective against *C. albicans* and *C. krusei* and showed low activity against *C. tropicalis* (Table 2). More interestingly, both strains were isolated from the same colony but from independent workers (Table 1). Because lower MICs were obtained for the extract of *Streptomyces* sp. TD025, this strain was selected to verify the chemical compounds responsible for the antimicrobial activity.

### 3.3. Bioactive Compounds of *Streptomyces* sp. TD025

**Figure 2:**
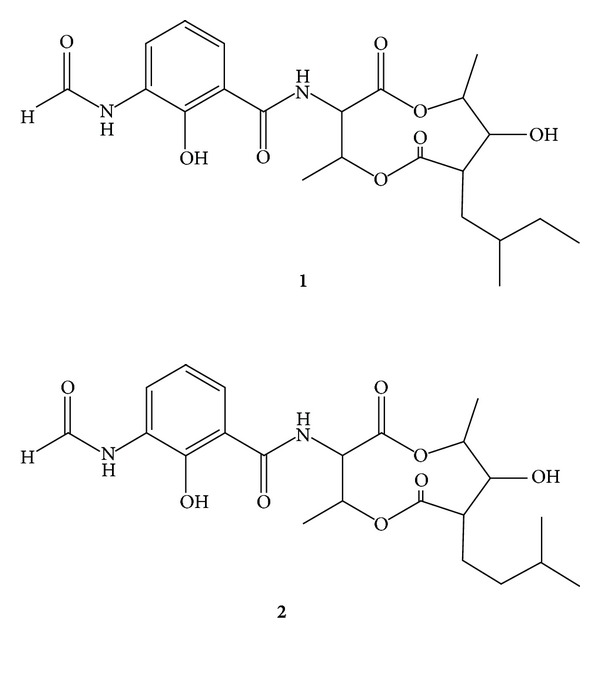
Chemical structures of compounds isolated from *Streptomyces* sp. TD025. (**1**) urauchimycin A; (**2**) urauchimycin B.

Chromatographic procedures revealed that EtOAc extract from TD025 contains two compounds (**1** and **2**, Figure 2). Compounds **1** and **2** exhibited typical NMR data of urauchimycins (Figures S1 and S2). Their NMR data are in agreement with those previously reported by Imamura et al. [46]. Although these urauchimycins (**1** and **2**) have already been isolated, they have never been tested on various species of *Candida* as carried out in the present study.

The ^13^C NMR spectrum of **1** showed 22 carbon signals: four carbonyls (*δ* 179.0, *δ* 170.6, *δ* 169.8, and *δ* 158.7), three quaternary sp^2^ carbons (*δ* 150.6, *δ* 127.4, and *δ* 112.8), three methine aromatic carbons (*δ* 124.8, *δ* 120.6, and *δ* 119.0), two sp^3^ methylene group (*δ* 35.6 and *δ* 30.5), three sp^3^ methine groups (*δ* 54.0, *δ* 50.1, and *δ* 32.4), three oxymethinic groups (*δ* 77.1, *δ* 76.3, and *δ* 70.9), and four methyl groups (*δ* 18.4, *δ* 18.4, *δ* 15.1, and *δ* 11.4). Two carboxylic carbons *δ* 170.6 and *δ* 173.9 showed correlations with different hydrogens of the structure, showing a dilactone system of nine members, typical of the antimycin class.

The ^1^H NMR spectrum showed a singlet at *δ* 8.50, assigned to a hydrogen bounded to a carbonyl group and three aromatic hydrogens at *δ* 8.55 (dd, *J* 8.1 and 1.2 Hz), *δ* 7.24 (dd, *J* 8.1 and 1.2 Hz), and d 6.92 (t, *J* 8.1 Hz), suggesting a 1,2,3 trisubstituted aromatic ring. The substance was identified as urauchimycin A by comparison with the literature data [46].

The ^1^H and ^13^C NMR spectram of **2** were very similar to those observed for compound **1**. Differences were observed in chemical shifts of the hydrogen of the methyl and methylene groups of the side chain. Based on published data [46] compound **2 **was identified as Urauchimycin B, an isomer of compound **1**.

Urauchimycins belong to the antimycin class, a group of well-known antifungals. Antimycins act by inhibiting the electron flow in the mitochondrial respiratory chain [47]. Antimycins have been previously identified in *Streptomyces *isolated from the integument of attine ants [32–34]. Schoenian and colleagues [32] detected the well-know antimycins A1–A4 in 50% of the actinobacteria identified as *Streptomyces* isolated from workers of several *Acromyrmex* species. These data along with the rare antimycins identified in the present study indicate that this chemical class is often produced by *Streptomyces *associated with attine ants. Compounds belonging to this class may have an important role in the attine ant-microbe association.

Another antifungal compound widely distributed in *Streptomyces* associated with attine ants is candicidin [31–34], which was not detected in *Streptomyces *sp. TD025. It is possible that candicidin was lost in one of the purification steps of the AcOEt extract or it is not produced by this strain. 

Urauchimycins A and B were previously isolated from *Streptomyces* sp. Ni-80 isolated from a marine sponge in Urauchicove, Irimore, Japan. These substances were the first antimycins having an odd number of carbons and a branching side chain [46]. Imamura et al. [46] suggested that such structures are the result of an evolution of actinobacteria in the marine environment, which could have resulted in a change in their secondary metabolism.

In 2006, two new urauchimycins were described: urauchimycin C, isolated from *Streptomyces* sp. B1751 from marine sediment, and urauchimycin D, isolated from *Streptomyces* sp. AdM21 from soil [48]. In the study by Imamura and coworkers [46], the urauchimycins A and B inhibited the morphological differentiation of *C. albicans* up to a concentration of 10 *µ*g · mL^−1^. Urauchimycins C and D showed no inhibitory activity against *C. albicans*, *Mucor miehei*, and bacteria [48].

The study of antimicrobial activity of urauchimycins A and B was restricted to *C. albicans* in the work by Imamura and colleagues [46]. The reisolation of these molecules in the present study allowed a better evaluation of its spectrum of activity. The urauchimycins from *Streptomyces* sp. TD025 presented MIC values equivalent to the reference antifungal nystatin for *C. albicans* and *C. glabrata* (Table 3). Urauchimycin B showed inhibitory activity against all *Candida* strains evaluated, showing MIC similar to those provided by nystatin.

Urauchimycin B showed a broad spectrum of activity against *Candida* spp. with MIC values equivalent to the antifungal nystatin, which indicates the potential for medical use. For many years, antimycins were used for the treatment of human infections, but due to its mechanism of action and associated side effects, its use in human treatment was discontinued [47]. However, with the pressing need for new antifungal agents that complement or substitute for the scarce products available on the market, it is interesting and necessary to determine the toxicity presented by urauchimycin B, to assess whether it can be used as an antifungal agent for humans and animals. In addition, evaluation of the isolated compound against *Candida* species resistant to commercially available antifungal agents should be performed to confirm the potential of this relatively unexplored antifungal. 

Here we show that *Trachymyrmex* ants, one attine genus understudied with respect to its microbial symbionts, harbor antimicrobial-producing actinobacteria. As observed by other authors [28–33], the present study demonstrates that actinobacteria of attine ants are able to produce antifungal compounds active against other fungal species and not only against the specific fungal parasite *Escovopsis*.

Moreover, our study corroborates previous work [35] that suggests the attine ant-microbe association is a promising source of microorganisms that produce active metabolites. The few recent studies that focused on the chemical characterization of bioactive compounds produced by actinobacteria associated with attine ants support the potential isolation of novel molecules with biological activity [28–32]. Thus, an exploration program of isolation of bioactive molecules from actinobacteria from attine ants certainly will result in the discovery of novel compounds with activity against microorganisms that are potentially pathogenic to humans.

## 4. Conclusion

As suggested by Poulsen [35], we found that the integument of *Trachymrymex* ants is a potential source for the isolation of actinobacteria that produce bioactive molecules. The isolation of Urauchimycins A and B enabled, for the first time, the evaluation of their activity against various *Candida* species. Urauchimycin B showed a broad spectrum of activity and MIC values equivalent to the reference antifungal nystatin. Toxicity studies and *in vivo* activity should be carried out in order to verify the potential use of this molecule in the treatment of fungal infections.

## Supplementary Material

Table S1: Actinobacteria strains used in the present study.
Figure S1: ^1^H NMR of Urauchimycin A (CDCl_3_, 400 MHz).
Figure S2: ^1^H NMR of Urauchimycin B (CDCl_3_, 400 MHz).

## Figures and Tables

**Table 1:** Actinobacteria identification according to 16S rDNA sequencing.

Isolate Id	bp^1^	NCBI-GenBank closest relative	Coverage	%	Accession #
TD016	1251	*Nocardia neocaledoniensis* DSM 44717	100	100	JF797311
TD017	1248	*Nocardia neocaledoniensis* DSM 44717	100	100	JF797311
TD018	1184	*Streptomyces zaomyceticus* NRLL B-2038	99	100	NR044144
TD019	1342	*Streptomyces alivochromogenes* NBRC 3404	99	100	AB184761
TD020	1167	*Streptomyces sannanensis* NBRC 14239	99	99	NR041160
TD021	1250	*Streptomyces mauvecolor* NBRC 13854	100	99	NR041154
TD022	1254	*Streptomyces lydicus* CGMCC 4.1412	100	100	JN566018
TD023	1246	*Streptomyces* sp. QZGY-A17	100	100	JQ812074
TD025	1333	*Streptomyces* sp. QLS92	100	99	JQ838121
		*Streptomyces cirratus *	100	99	JQ222143
TD027	1342	*Streptomyces alivochromogenes* NBRC 3404	99	100	AB184761
TD028	1353	*Actinoplanes ferrugineus *	100	99	AB048221
TD030	1255	*Streptomyces* sp. CA13	100	99	AB622252
TD032	1263	*Streptomyces chartreusis* NBRC 12753	100	100	NR041216
TD033	1278	*Streptomyces griseoplanus *	99	100	HQ699516
TD034	1283	*Amycolatopsis decaplanina* DSM 44594	100	100	NR025562
TD035	1320	*Streptomyces atriruber* NRLL B-24676	100	99	FJ169330
TD045	1183	*Amycolatopsis equina *	100	99	HQ021204
TD047	1173	*Amycolatopsis albidoflavus* NBRC100337	100	100	AB327251
TD049	1260	*Streptomyces luteogriseus* NBRC 13402	100	99	NR041128
TD050	1261	*Streptomyces rubiginosohelvolus* NBRC 12912	100	100	NR041093
TD051	1173	*Amycolatopsis albidoflavus* NBRC100337	100	100	AB327251
TD053	1265	*Streptomyces kunmingensis* NBRC14463	99	98	AB184597
TD055	1173	*Amycolatopsis albidoflavus* NBRC100337	100	100	AB327251
TD058	1257	*Streptomyces globisporus* KCTC 9026	100	100	HQ995504

^1^bp: base pair.

**Table 2:** Minimum inhibitory concentrations (*μ*g·mL^−1^) of actinobacteria extracts towards different medically important *Candida* species.

Isolate ID	*C. albicans *	*C. dubliniensis *	*C. glabrata *	*C. krusei *	*C. parapsilosis *	*C. tropicalis *
	CBS 562	CBS 7987	CBS 138	CBS 573	CBS 604	CBS 94
TD016	∗	∗	∗	∗	∗	∗
TD017	∗	∗	∗	∗	∗	∗
TD018	∗	∗	∗	∗	∗	∗
TD019	60	900	∗	40	80	1000
TD020	∗	∗	∗	∗	∗	∗
TD021	∗	∗	∗	∗	∗	∗
TD022	800	∗	1000	∗	∗	∗
TD023	∗	∗	∗	∗	∗	∗
TD025	40	700	∗	15	125	1000
TD027	60	1000	∗	15	200	∗
TD028	∗	∗	∗	∗	∗	∗
TD030	∗	∗	∗	∗	∗	∗
TD032	1000	∗	∗	∗		∗
TD033	10	800	∗	125	200	∗
TD034	500	∗	∗	∗		∗
TD035	∗	∗	∗	∗	∗	∗
TD045	∗	∗	∗	∗	∗	∗
TD047	∗	∗	∗	∗	∗	∗
TD049	∗	∗	∗	∗	∗	∗
TD050	∗	∗	∗	∗	∗	∗
TD051	∗	∗	∗	∗	∗	∗
TD053	∗	∗	∗	∗	∗	∗
TD055	∗	∗	∗	∗	∗	∗
TD058	∗	∗	∗	∗	∗	∗

*Minimum inhibitory concentration > 1000 *μ*g·mL^−1^.

**Table 3:** Minimum inhibitory concentration (MIC) and minimum fungicide concentrations (MFC) (*μ*g·mL^−1^) of Urauchimycins A and B obtained from *Streptomyces *sp. TD025 in comparison with the antifungal Nystatin.

Candida species	Urauchimycin A		Urauchimycin B		Nystatin	
	MIC	MFC	MIC	MFC	MIC	MFC
*C. albicans *	1	∗	1	3	1	2
*C. dubliniensis *	800	∗	2	3	1	2
*C. glabrata *	2	15,6	2	2	1	1
*C. krusei *	15,6	15,6	2	3	2	3
*C. parapsilosis *	∗	∗	2	2	1	2
*C. tropicalis *	∗	∗	2	2	4	4

*>1000 *μ*g·mL^−1^.
