# Supplementary material for: Anti-Candida Properties of Urauchimycins from Actinobacteria Associated with Trachymyrmex Ants
Source: Biomed Res Int. 2013 Mar 18;2013:835081. doi: 10.1155/2013/835081 (PMC3613088; doi:10.1155/2013/835081)

SUPPLEMENTARY MATERIAL

Submitted to *BioMed Research International* - Special Issue "Actinomycetes: Role in Biotechnology and Medicine"

Anti-*Candida* properties of urauchimycins from actinobacteria associated with *Trachymyrmex* ants

Thais D. Mendes, Warley S. Borges, Andre Rodrigues, Scott E. Solomon, Paulo C. Vieira, Marta C. T. Duarte, Fernando C. Pagnocca

Contents:

Table S1. Actinobacteria strains used in the present study.

Figure S1. ^{1}H NMR of Urauchimycin A ($CDCl_3$, 400 MHz).

Figure S2. ^{1}H NMR of Urauchimycin B ($CDCl_3$, 400 MHz).

Table S1. Actinobacteria strains used in the present study

Colony Id	Worker[1]	Isolate Id	Ant	Collection site	GPS
CTL080820-02	A	TD016	*Trachymyrmex* sp.	Estação Ecológica do Panga, Uberlândia – MG	S 19°17.291’ W 48°39.670’
CTL080820-02	B	TD017	*Trachymyrmex* sp.	Estação Ecológica do Panga, Uberlândia – MG	S 19°17.291’ W 48°39.670’
ARTD080903-01	B	TD018	*Trachymyrmex* sp.	UNESP, Rio Claro – SP[2]	S 10° 23.766’ W 48° 21.700’
ARTD080903-02	A	TD019	*Trachymyrmex* sp.	UNESP, Rio Claro – SP	S 10° 23.766’ W 48° 21.700’
ARTD080903-02	B	TD020	*Trachymyrmex* sp.	UNESP, Rio Claro – SP	S 10° 23.766’ W 48° 21.700’
ARTD080903-02	C1	TD021	*Trachymyrmex* sp.	UNESP, Rio Claro – SP	S 10° 23.766’ W 48° 21.700’
ARTD080903-02	C2	TD022	*Trachymyrmex* sp.	UNESP, Rio Claro – SP	S 10° 23.766’ W 48° 21.700’
ARTD080903-02	C3	TD023	*Trachymyrmex* sp.	UNESP, Rio Claro – SP	S 10° 23.766’

Colony Id	Worker[1]	Isolate Id	Ant	Collection site	GPS
					W 48° 21.700’
ARTD080903-03	A	TD025	*Trachymyrmex* sp.	UNESP, Rio Claro – SP	S 10° 23.766’<br>W 48° 21.700’
ARTD080903-03	D	TD027	*Trachymyrmex* sp.	UNESP, Rio Claro – SP	S 10° 23.766’<br>W 48° 21.700’
ARTD080903-04	A	TD028	*Trachymyrmex* sp.	UNESP, Rio Claro – SP	S 10° 23.766’<br>W 48° 21.700’
ARTD080903-04	B	TD030	*Trachymyrmex* sp.	UNESP, Rio Claro – SP	S 10° 23.766’<br>W 48° 21.700’
SES080909-08	A	TD032	*Trachymyrmex* sp.	Fazenda São Bento, Miranda – MS	S 19° 50.146’<br>W 57° 01.016’
SES080911-04	A1	TD033	*Trachymyrmex* sp.	Fazenda São Bento, Miranda – MS	S 19° 57.612’<br>W 56° 99.019’
SES080911-04	A2	TD034	*Trachymyrmex* sp.	Fazenda São Bento, Miranda – MS	S 19° 57.612’<br>W 56° 99.019’
SES080911-06	A	TD035	*Trachymyrmex* sp.	Fazenda São Bento, Miranda – MS	S 19° 49.474’<br>W 56° 01.079’
SES080921-03	A	TD045	*Trachymyrmex* sp.	Estação Ecológica do Panga, Uberlândia – MG	S 19°17.291’<br>W 48°39.670’
SES080921-03	B	TD047	*Trachymyrmex* sp.	Estação Ecológica do Panga, Uberlândia – MG	S 19°17.291’<br>W 48°39.670’
SES080922-03	C	TD049	*Trachymyrmex* sp.	Estação Ecológica do Panga, Uberlândia – MG	S 19°17.291’<br>W 48°39.670’
SES080922-03	D	TD050	*Trachymyrmex* sp.	Estação Ecológica do Panga, Uberlândia – MG	S 19°17.291’<br>W 48°39.670’
SES080924-01	A1	TD051	*Trachymyrmex* sp.	Estação Ecológica do Panga, Uberlândia – MG	S 19°17.291’<br>W 48°39.670’
SES080924-01	A2	TD053	*Trachymyrmex* sp.	Estação Ecológica do Panga, Uberlândia – MG	S 19°17.291’<br>W 48°39.670’
SES080924-01	B	TD055	*Trachymyrmex* sp.	Estação Ecológica do Panga, Uberlândia – MG	S 19°17.291’

Colony Id	Worker[1]	Isolate Id	Ant	Collection site	GPS
					W 48°39.670’
SES080924-02	B	TD058	*Trachymyrmex* sp.	Estação Ecológica do Panga, Uberlândia – MG	S 19°17.291’ W 48°39.670’

[1] Letters refers to an ant worker from which we isolated actinobacteria. Letters followed by numbers indicate that more than one actinobacteria was isolated from the same worker.

[2] UNESP: São Paulo State University; SP: São Paulo State; MS: Mato Grosso do Sul State; MG: Minas Gerais State

Fig. S1.

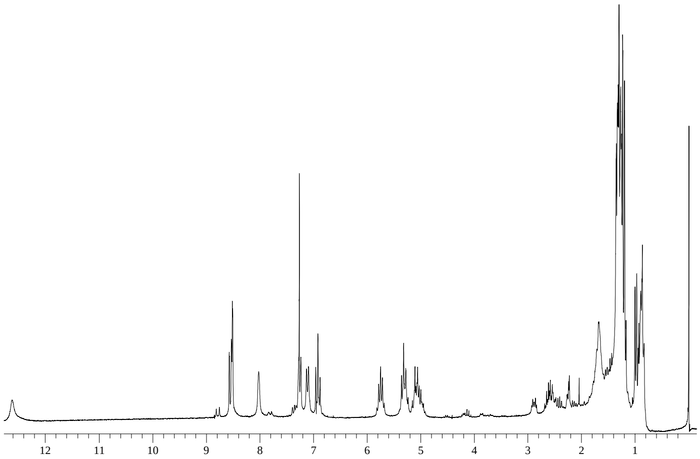

Fig. S2.

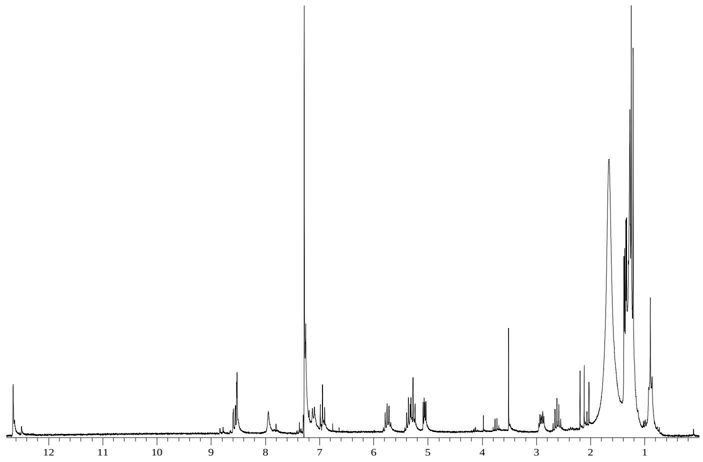

Supplement: Supplementary file 1 — Table S1: Actinobacteria strains used in the present study. Figure S1: 1H NMR of Urauchimycin A (CDCl3, 400 MHz). Figure S2: 1H NMR of Urauchimycin B (CDCl3, 400 MHz). [file 835081.f1.pdf]
